# Sensitivity enhancement of flexible gas sensors via conversion of inkjet-printed silver electrodes into porous gold counterparts

**DOI:** 10.1038/s41598-017-09174-5

**Published:** 2017-08-21

**Authors:** Yunnan Fang, Mitra Akbari, Jimmy G. D. Hester, Lauri Sydänheimo, Leena Ukkonen, Manos M. Tentzeris

**Affiliations:** 1 School of Materials Science and Engineering, Georgia Institute of Technology, Atlanta, GA 30332-0245 USA; 2 Department of Electronics and Communications Engineering, Tampere University of Technology, 33101 Tampere, Finland; 3 School of Electrical and Computer Engineering, Georgia Institute of Technology, Atlanta, GA 30332-0250 USA

## Abstract

This work describes a facile, mild and general wet chemical method to change the material and the geometry of inkjet-printed interdigitated electrodes (IDEs) thus drastically enhancing the sensitivity of chemiresistive sensors. A novel layer-by-layer chemical method was developed and used to uniformly deposit semiconducting single-wall carbon nanotube (SWCNT)-based sensing elements on a Kapton^®^ substrate. Flexible chemiresistive sensors were then fabricated by inkjet-printing fine-featured silver IDEs on top of the sensing elements. A mild and facile two-step process was employed to convert the inkjet-printed dense silver IDEs into their highly porous gold counterparts under ambient conditions without losing the IDE-substrate adhesion. A proof-of-concept gas sensor equipped with the resulting porous gold IDEs featured a sensitivity to diethyl ethylphosphonate (DEEP, a simulant of the nerve agent sarin) of at least 5 times higher than a similar sensor equipped with the original dense silver IDEs, which suggested that the electrode material and/or the Schottky contacts between the electrodes and the SWCNTs might have played an important role in the gas sensing process.

## Introduction

A number of metals, such as copper (Cu)^1,2^, nickel (Ni)^3^, silver (Ag)^4^ and gold (Au)^5,6^, have been implemented in the form of nanoparticle-based inkjet inks and used to inkjet-print various electronic structures or devices. Among these metals, Cu, Ag and Au all tarnish to a greater or lesser extent^7^. It is worth mentioning that Ag is the best conductor of heat and electricity^8^ and silver nanoparticle (SNP)-based inks represent the most important commercial nanotechnology-derived product and are the most commonly used, most widely studied and with a well-settled technology. SNP inks also have the highest sales volume among all metallic inks^9^. However, Ag, especially nanoparticle Ag, is the metal which most readily tarnishes even under dry and ambient conditions, due to the presence of pollutants like hydrogen sulphide and carbonyl sulphide in air^10,11^, which leads to the significant reduction of its conductivity^12^. When inkjet-printed Ag structures/devices are used in aqueous environments, for example when used as electrodes in electrochemical devices, their poor conductivity caused by oxidation and degradation under an applied electrical potential can be a major concern^6^. Consequently, some passivation treatments, such as deposition of nickel^13^ or polymers^14^ on the Ag electrodes, have been employed in the past to prevent or reduce their tarnishing. On the other hand, Au is the most malleable and ductile of all metals^8^, and one of the most inert and stable chemical elements, while in the meantime it is an excellent electricity conductor. For these reasons, Au is preferred to Ag for many applications. For example, printed circuit board (PCB) electrodes for medical use (such as those utilized for the high-resolution gastrointestinal electrical mapping) prefer Au to Ag, since low-temperature sterilization of the electrodes relies on oxidizing agents such as ozone and hydrogen peroxide^15^ which would oxidize Ag^16,17^. In another example, ZnO-based chemiresistive gas sensors with Ag electrodes exhibited not only lower sensitivity but also longer response and recovery times for both CO and NO_2_ than their counterparts with Au electrodes, due to the oxidation or the poisoning of Ag electrodes by the analyte gases^18^.

There has been increasing evidence showing that the geometry of the contacts between the metal electrodes and the semiconducting materials can radically change the device performance. The contact resistance that is formed in the electrode-semiconductor interface can sometimes significantly contribute to the response of the sensors^19,20^, although it has been conventionally ignored^21^. A Schottky barrier has been found in the contacts of semiconducting carbon nanotubes (CNTs) with Au^22^ and increasing the Schottky contact area between semiconducting CNTs and Au/Cr electrodes has been shown to radically increase the sensitivity of biosensors^23^.

Inkjet-printing with Au nanoparticle inks, however, has not been as common as with SNP inks. Compared with SNP-based inks, Au nanoparticle-based inks are prohibitively expensive and the techniques for preparing and inkjet-printing these inks are not as mature as those for SNP inks. In addition, the high sintering temperature for Au nanoparticle inks (>190 °C) has limited their applications to flexible electronic devices, since a number of commonly used flexible substrates (such as copy paper, filter paper, photo paper, polyethylene naphthalate (PEN) films, and polyethylene terephthalate (PET) films) have a maximum working temperature of <190 °C. On the other hand, the sintering temperature of SNP-based inks can be as low as 120 °C, which is compatible with most commonly used flexible substrates.

To facilitate reliable and relatively low-cost inkjet-printing of high-performing flexible electronic devices, it is desirable to take advantage of the excellent chemical stability of Au nanoparticles as well as the well-settled technology and the low sintering temperature of SNP inks. Furthermore, highly porous Au electrodes can create more Schottky contacts with a semiconducting sensing material than their dense counterparts, which is highly desired for sensing applications. One way to meet all these desires is to inkjet-print Ag traces on a substrate with an SNP ink, sinter the inkjet-printed Ag nanoparticles at a relatively low temperature to achieve the desired connectivity and conductivity, and then convert the resulting Ag patterns into their porous Au counterparts at a temperature that is safe for both the substrate and the adhesion between the metal traces and the substrate. There have been a few previous publications reporting a one-step process to convert Ag nanoparticles^24^ and Ag micro^25^-/nano^26^-structures into their Au counterparts under relatively harsh conditions (i.e., at 100 °C in an auric chloride (HAuCl_4_) aqueous solution). However, this process was not able to create pores in the resulting Au components. Additionally, when this high-temperature and in-solution conversion process is applied to Ag traces that have been inkjet-printed on a substrate, there is a serious concern whether the adhesion between the traces and the substrate can survive the relatively harsh process, especially when the substrate has a smooth surface (such as in the case of glass, silicon wafer, Kapton^®^ polyimide, PET and PEN films). In fact, it has been shown that the adhesion between inkjet-printed Ag interdigitated electrodes (IDEs) and a plastic substrate (PET or PEN) was not even able to survive exposure to buffers^6^, not to mention chemically converting the Ag traces to their Au counterparts in solutions. Apparently enhancing the trace-substrate adhesion and reducing the harshness of the conversion conditions (for example reducing the conversion temperature) are among the solutions to the aforementioned concern.

This work focused on enhancing the sensitivity of flexible chemiresistive sensors by changing the material and the porosity of their inkjet-printed IDEs without losing the IDE-substrate adhesion. First, a novel layer-by-layer chemical process was developed and used to uniformly deposit sensing elements (single-wall carbon nanotubes (SWCNTs) complexed with a chemoselective compound (selector)) on a relatively large piece (for example 95 mm × 95 mm) of Kapton^®^ polyimide film, a commonly utilized flexible substrate. Second, fine-featured Ag IDEs were inkjet-printed on the resulting surface-functionalized substrate to form an array of gas sensors. Finally, a facile and mild two-step process was employed to convert the Ag IDEs into their highly porous Au counterparts under ambient conditions without losing the adhesion between the IDEs and the substrate. Through these processes, the chemical stability (i.e., the chemical inertness, particularly the resistance to tarnishing) of the IDEs was enhanced, the contact area of the IDEs with the sensing elements was increased, and the risk of losing the IDE-substrate adhesion was minimized. In addition, among all the sensors fabricated on the same piece of substrate, the individual differences originated from the components other than the electrodes were minimized, thus allowing for a fair performance comparison between a sensor with the original dense Ag IDEs and one with the resulting highly porous Au IDEs.

To demonstrate the utility of this approach, sensors electroded with the original inkjet-printed dense Ag IDEs and with the converted porous Au IDEs were both exposed to diethyl ethylphosphonate (DEEP) vapour to compare their sensing behaviour. DEEP is a simulant of G-type nerve agent sarin, one of the most-toxic chemical weapons in existence. Sarin has been repeatedly used by rogue states and terrorist organizations against military troops or civilians causing mass casualties^27–29^, and thus the detection of sarin or its simulants is becoming more and more of a concern in the world. Our DEEP sensing trials showed that a sensor with the converted porous Au IDEs featured a sensitivity at least five times greater than a similar sensor with the original inkjet-printed dense Ag IDEs.

## Results

### Conversion of inkjet-printed Ag IDEs into porous Au counterparts

**Figure 1:**
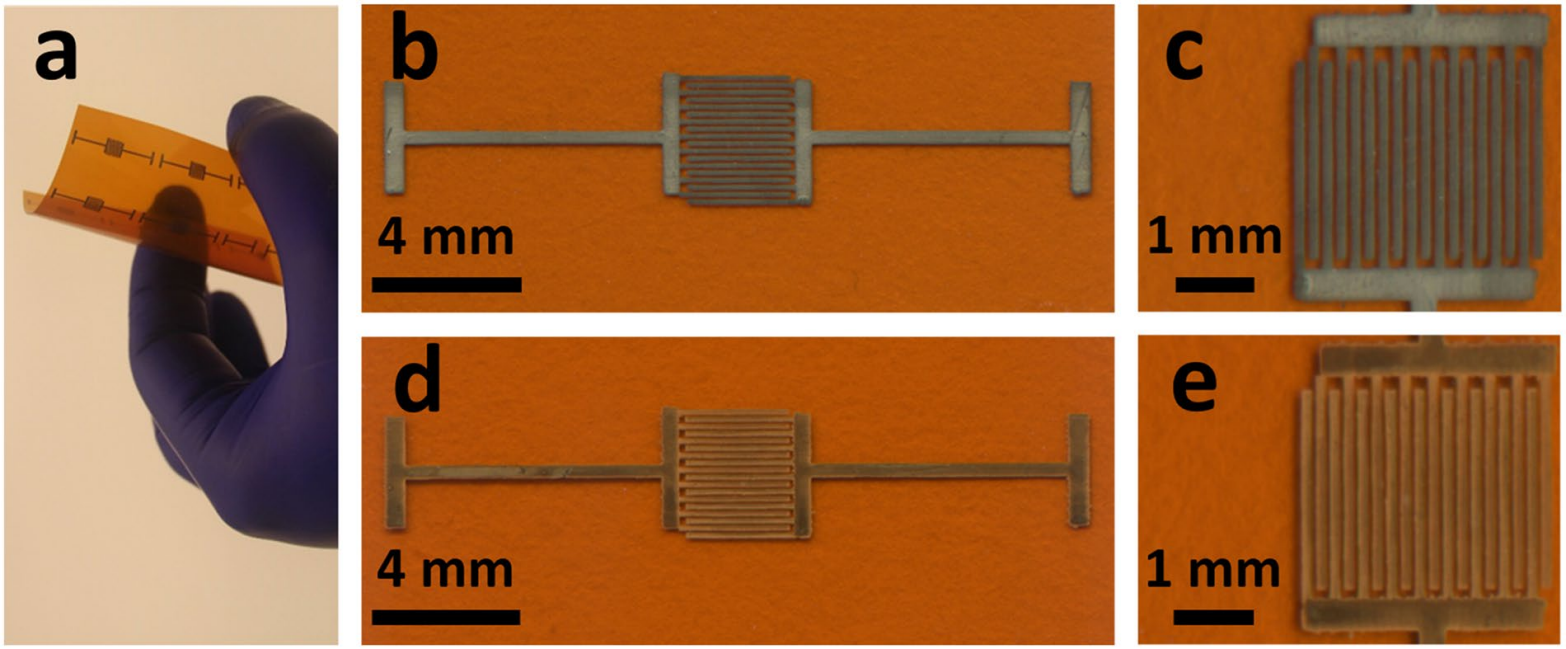
Scanned images of the inkjet-printed sensors before (**a**,**b** and **c**) and after (**d** and **e**) the conversion of the Ag IDEs into their Au counterparts.

**Figure 2:**
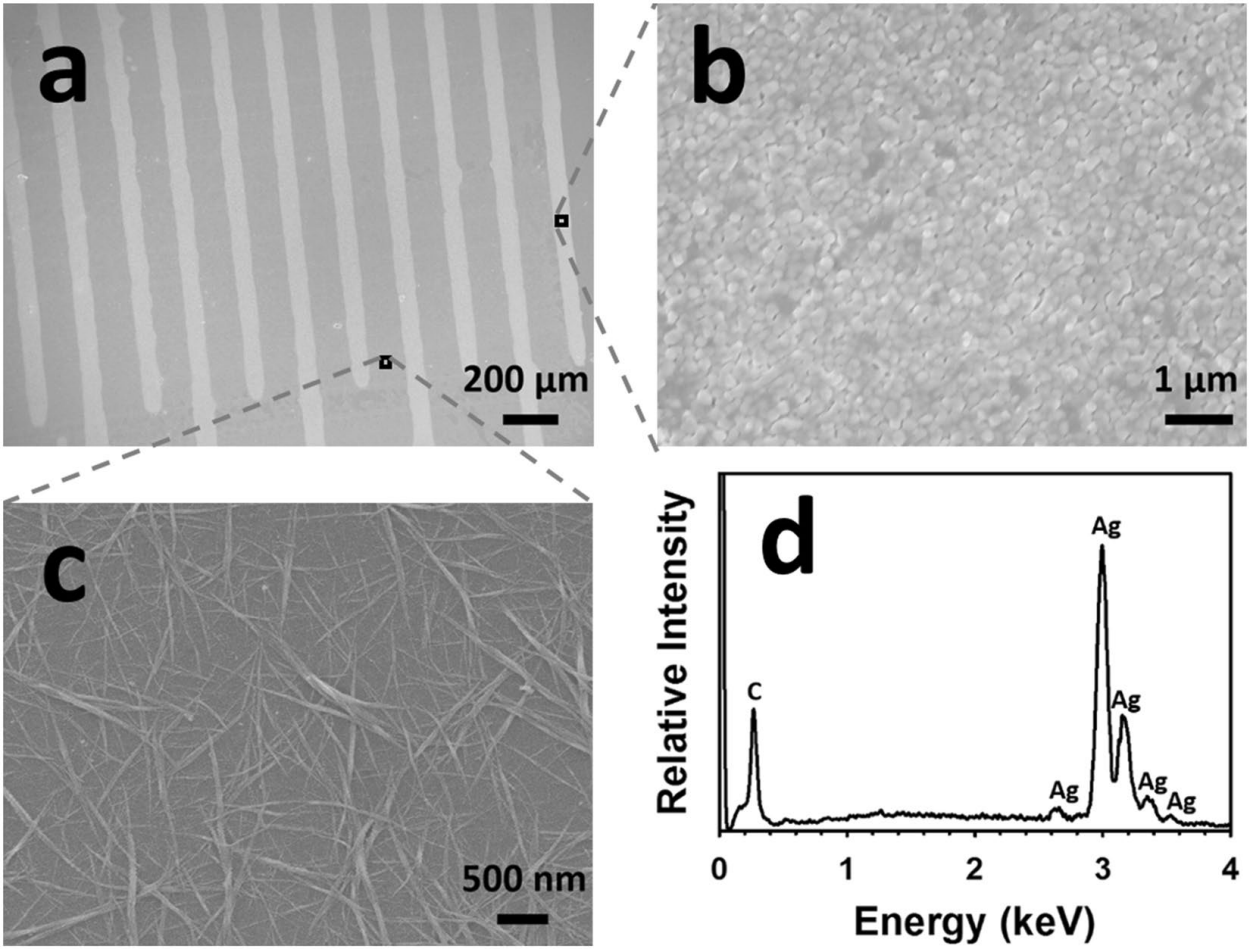
Scanning electron microscopy (SEM) and energy dispersive X-ray spectroscopy (EDX) analyses of a sensor before the Ag-to-Au conversion. (**a**) SEM image of the sensor; (**b**) SEM image focusing on an IDE finger of the sensor; (**c**) SEM image focusing on the SWCNT-selector complexes between two adjacent IDE fingers; (**d**) EDX pattern of the specimen shown in (**b**).

**Figure 3:**
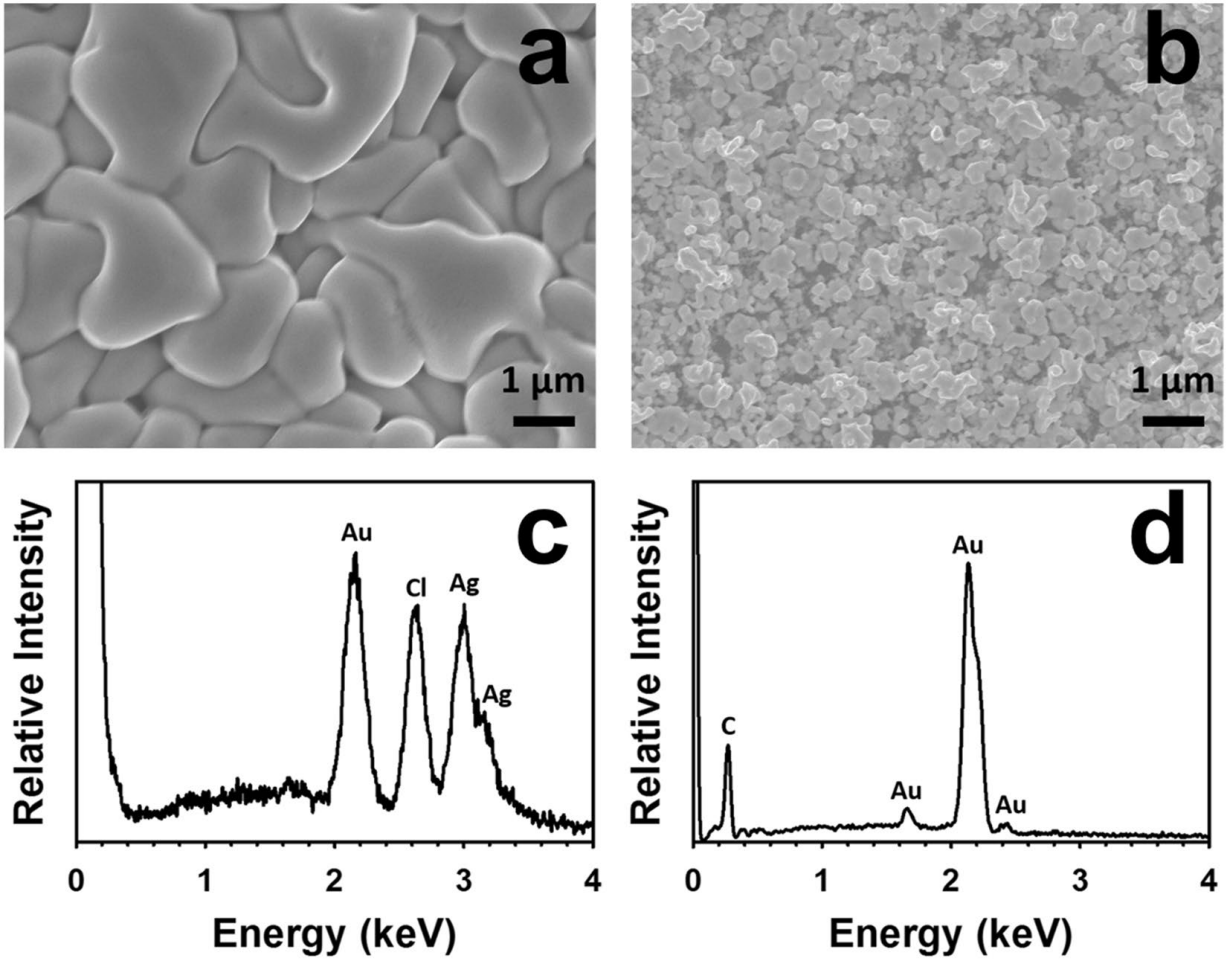
SEM images and EDX patterns of the IDEs of an SWCNT-based sensor at different stages after the Ag-to-Au conversion. (**a**) SEM image of the IDEs after the Ag-to-Au conversion but before the removal of the side product AgCl; (**b**) SEM image of the IDEs after the Ag-to-Au conversion and the removal of the side product AgCl; (**c**) and (**d**) EDX patterns of the specimens shown in (**a**) and (**b**), respectively.

**Figure 4:**
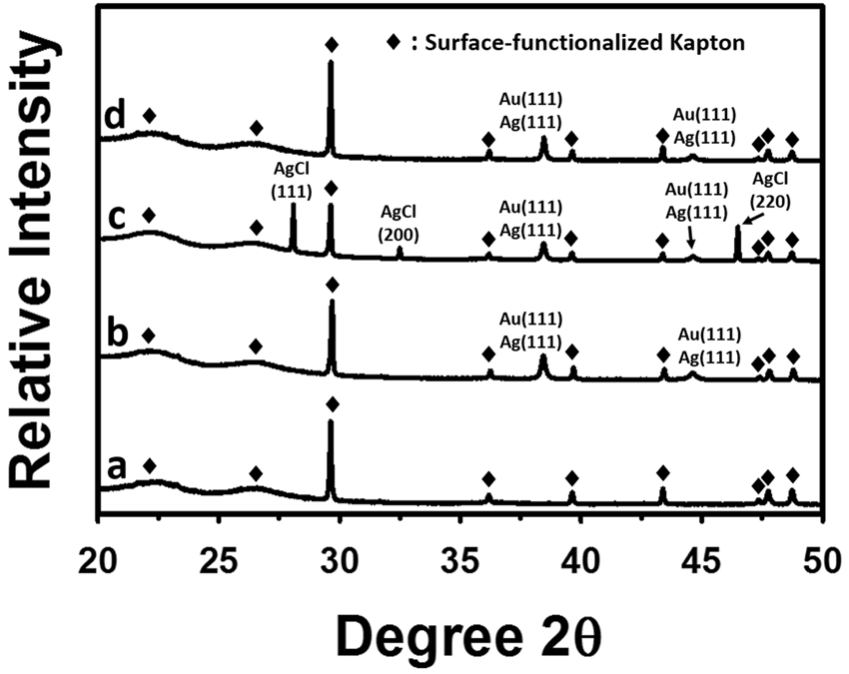
X-ray diffraction (XRD) analyses of (**a**) the starting Kapton^®^ substrate (functionalized with the SWCNT-selector complexes), (**b**) the inkjet-printed Kapton^®^-Ag structure after the annealing but before the Ag-to-Au conversion, (**c**) the Kapton^®^-IDE structure after the Ag-to-Au conversion but before the selective removal of the AgCl, and (**d**) The Kapton^®^-IDE structure after the selective removal of the AgCl.

A piece of Kapton^®^ HN substrate was cleaned and functionalized with SWCNT-based sensing elements, and then an array of flexible sensor prototypes was fabricated on the substrate by inkjet-printing Ag IDEs with an SNP ink on top of the sensing elements. An optical image of the array of sensors is shown in Fig. 1a. Scanned images at low and high magnifications of a single sensor prototype before the Ag-to-Au conversion are shown in Fig. 1b and c, respectively. After converting the inkjet-printed Ag IDEs of the sensor (as shown in Fig. 1b,c) into their Au counterparts through a wet chemical reaction with HAuCl_4_, the resulting Au IDEs were morphologically similar to the starting Ag IDEs under low magnifications (Fig. 1d,e), but the Ag and the Au IDEs had different colours. Figure 2 shows the scanning electron microscopy (SEM) images of a sensor (before the Ag-to-Au conversion) and the energy dispersive X-ray spectroscopy (EDX) pattern of its Ag IDEs. The inkjet-printed Ag particles (after annealing at 120 °C for 3 hours) were basically spherical with a diameter of around 150 nm and densely packed (Fig. 2b). As shown in Fig. 2c, between two adjacent IDE fingers there were SWCNT particles (functionalized with the selector 2-(2-hydroxy-1, 1, 1, 3, 3, 3-hexafluoropropyl)-1-naphthol) that were randomly and evenly distributed on the Kapton^®^ substrate. The EDX analysis (Fig. 2d) of the Ag IDEs shows the element Ag as well as the element C (Note: the C peak in the Fig. 2d originated from the carbon-containing Kapton^®^ substrate and the carbon film that was sputter-coated on the sensor in order to make the sample conductive for SEM and EDX analyses). After the incubation with the HAuCl_4_ solution, there was a significant change in the morphology of the resulting IDEs: the particles became much larger than the starting inkjet-printed Ag nanoparticles and their shape turned irregular (Fig. 3a). The EDX analysis of the resulting IDEs shows the presence of the elements Au, Ag and Cl (Fig. 3c). The subsequent incubation with a solution of saturated NaCl brought another significant change in the morphology of the resulting IDEs: the particles drastically shrank and became loosely packed, which resulted in highly porous IDEs (Fig. 3b). The EDX analysis of the resulting porous IDEs shows the disappearance of the elements Ag and Cl and the retaining of the element Au (Fig. 3d) (Again, the C peak in EDX pattern in Fig. 3d originated from the carbon-containing Kapton^®^ substrate and the carbon film that was sputter-coated on the sensor to meet the conductivity requirement of SEM and EDX analyses). X-ray diffraction (XRD) patterns of the Kapton^®^-IDE structure at different stages are shown in Fig. 4. It is worth mentioning that the diffraction peaks of Au and Ag overlapped in the 2θ range scanned in this work^30,31^, and as a result, the X-ray diffraction analyses of the structure (Fig. 4) were not able to differentiate Au from Ag. However, when combining the X-ray diffraction patterns (Fig. 4) with the EDX patterns of the IDEs at different stages (Figs 2d and 3c,d), it can be concluded that (1) the IDEs before the Ag-to-Au conversion were made of Ag (Figs 2d and 4b), (2) the incubation with the HAuCl_4_ solution led to the conversion of Ag into Au and AgCl (Figs 3c and 4c), and (3) another incubation with the saturated NaCl solution resulted in the selective dissolution of AgCl leaving only Au behind (Figs 3d and 4d). Based on these results, we can conclude that our two-step room temperature process has converted the starting inkjet-printed dense Ag IDEs into their porous Au counterparts.

### Adhesion assessment

The adhesion between the dense Ag or the porous Au IDEs and the Kapton^®^ substrate was assessed by visual inspection and Scotch-tape peel tests. Visual inspection showed that both the dense Ag and the porous Au IDEs adhered well to the Kapton^®^ substrate and no detachment was observed, indicating that the IDE-substrate adhesion was not lost after the dense Ag IDEs had been converted into the porous Au IDEs. Scotch-tape peel tests were performed to assess the strength of the adhesion of both the Ag and the Au IDEs to the Kapton^®^ substrate. Sensors with the original dense Ag IDEs and with the converted porous Au IDEs were first stuck to the adhesive side of a piece of Scotch^®^ tape (Supplementary Figure S1a,c), and then the tape was slowly peeled off from the sensors at an angle of ~90°. As shown in Supplementary Figure S1b, after the tape had been peeled off, the dense Ag IDEs on the Kapton^®^ substrate were basically still intact (there was only one tiny Ag particle with a diameter of ~0.2 mm transferred to the tape). By contrast, the Au IDEs seemed delaminated by the tape: quite a lot of top-layer Au residues on the Kapton^®^ substrate were transferred to the tape after the tape had been removed from the Au IDEs (actually the Au residues on the tape essentially formed an intact IDE pattern) (Supplementary Figure S1d), while the Au IDEs that remained on the Kapton^®^ substrate still appeared intact (Supplementary Figure S1d). By visual inspection, the adhesion between the Au IDEs and the substrate seemed unaffected by the peeling-off of the tape.

### Gas sensing with SWCNT-based sensors electroded with dense Ag and with porous Au IDEs

Proof-of-concept sensor prototypes with the dense Ag IDEs and with the converted porous Au IDEs were both exposed to 2.0 ppm DEEP vapour and their resistance changes were monitored and recorded in time, with our homemade sensing system automated with NI LabVIEW software. The relative sensitivity S is defined by the formula

$$S=\frac{R-{R}_{0}}{{R}_{0}}$$

where R_0_ and R are the resistance values of a sensor right before and at a particular time after, respectively, the sensor was exposed to the target vapour.

**Figure 5:**
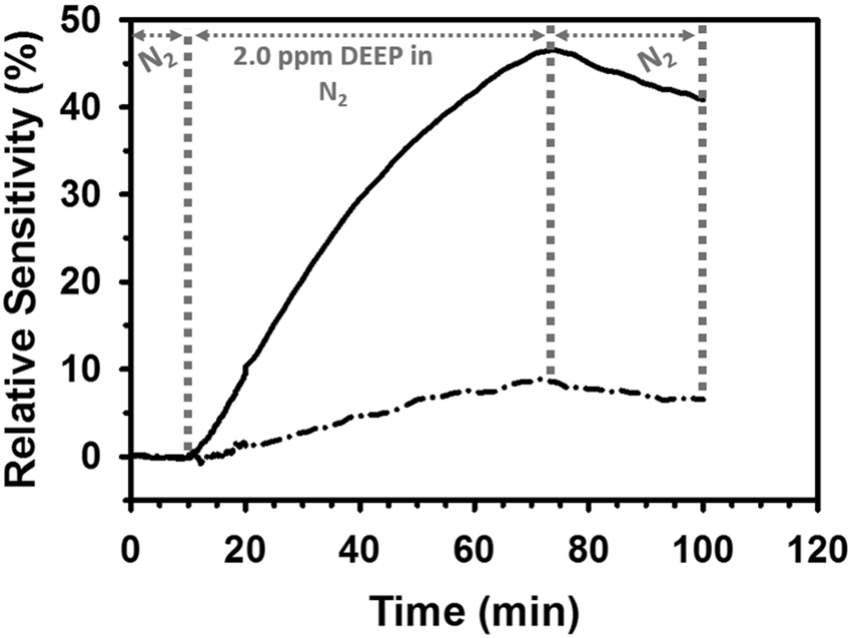
Typical sensing behavior of the proof-of-concept SWCNT-based sensors with dense Ag IDEs (dash-dot line) and with porous Au IDEs (solid line) upon exposure to 2.0 ppm DEEP vapour.

As shown in Fig. 5, both the sensor with the original inkjet-printed dense Ag IDEs and the sensor with the converted porous Au IDEs were virtually non-responsive to the carrier gas nitrogen (0–10 min time range). Upon the onset of 2.0 ppm DEEP vapour, the resistance of both sensors began to increase and continued increasing during the duration of DEEP release. In response to exposure to the DEEP vapour (10–73 min time range), the sensor with the porous Au IDEs changed its resistance much faster than its counterpart with the dense Ag IDEs. The sensor with the inkjet-printed Ag IDEs and with the converted porous Au IDEs featured a relative sensitivity (S) of ~9% (dash-dot line in Fig. 5) and ~47% (solid line in Fig. 5), respectively, after 63 min exposure to 2.0 ppm DEEP vapour. In other words, the conversion of the original inkjet-printed Ag IDEs into their porous Au counterparts increased the sensor sensitivity by more than 5 times.

## Discussion

The chemical reaction responsible for the one-step conversion of Ag nanostructures into their Au counterparts (without creating extra pores) at an elevated temperature (100 °C) has been shown to be as follows^24,26^:

1 $$3{\rm{Ag}}({\rm{s}})+{{\rm{HAuCl}}}_{4}({\rm{aq}})=\, > {\rm{Au}}({\rm{s}})+3{\rm{AgCl}}({\rm{aq}})+{\rm{HCl}}({\rm{aq}})$$

Under the experimental conditions, the AgCl formed in the reaction was shown to be completely soluble in water. Based on the EDX and XRD analyses of the IDEs at different stages (i.e., before and after the incubation with HAuCl_4_, and after the incubation with NaCl), we believe that the reaction responsible for our room temperature conversion process is as follows (similar to Reaction 1 except that the AgCl formed in the reaction was in solid form):

2 $$3{\rm{Ag}}({\rm{s}})+{{\rm{HAuCl}}}_{4}({\rm{aq}})=\, > {\rm{Au}}({\rm{s}})+3{\rm{AgCl}}({\rm{s}})+{\rm{HCl}}({\rm{aq}})$$

It is important for the AgCl in Reaction 2 to be in solid form and mixed with Au, since the creation of the pores in the Au IDEs was mainly due the selective removal of the side product AgCl. The size difference between an Ag atom (with a Van der Waals radius of 172 pm) and an Au atom (with a Van der Waals radius of 166 pm) contributed as well to the pore creation in the Au IDEs: when an Ag atom is converted into an Au atom, there is a slight shrinkage in size. Due to the fact that the converted porous Au IDEs were on a Kapton^®^ substrate, it was difficult to experimentally determine their porosity. However, by assuming the starting inkjet-printed Ag IDEs were fully dense, a theoretical rough estimation of the porosity of the Au IDEs can be made based on some inherent properties of Au and Ag and the stoichiometry shown in Reaction 2. The total pore volume of the porous Au IDEs can be roughly considered to result from the pore volume created by the dissolution of AgCl and from the shrinkage of the solid volume when three moles of Ag were converted into one mole of Au (Reaction 2). Using the densities of Au and Ag (19.32 g/cm^3^ and 10.49 g/cm^3^ for Au and Ag, respectively) and their atomic masses (196.97 and 107.87 for Au and Ag, respectively), it can be calculated that the porosity of the porous Au IDEs was about 67%.

The Scotch-tape peel tests with porous Au IDEs showed that the Au IDEs on the Kapton^®^ substrate delaminated when the tape was peeled off: only the top layer of the Au particles was peeled and transferred from the substrate to the Scotch^®^ tape, and the bottom layer seemed unaffected (Supplementary Figure S1d). This means that even though the adhesion between the porous Au IDEs and the Kapton^®^ substrate survived our two-step mild process that converted the dense Ag IDEs into their porous Au counterparts, the connection between the Au particles was apparently weakened by the creation of pores in the IDEs.

When inkjet-printed structures/devices have to be exposed to relatively harsh environments (such as aqueous or organic solvent-based solutions, bending, vibration *etc*.), the adhesion between the inkjet-printed traces and the substrate has been a constant concern. In this work, a novel, mild and facile method has been developed to convert inkjet-printed dense Ag IDEs of SWCNT-based gas sensors into highly porous Au IDEs without losing the IDE-substrate adhesion. Considering the fact that the Kapton^®^ HN substrate features a very smooth surface (with an arithmetic mean surface roughness of 0.67 nm^4^), the substrate surface functionalization performed in this work and the mild nature of the Ag-to-Au conversion were thus proved to be efficient in protecting the IDE-substrate adhesion.

The mechanism responsible for the sensitivity enhancement as a result of changing the material and the geometry of the electrodes is still not clear. Based on our experimental observations and the limited relevant literature, we speculated that the significant sensitivity enhancement was due to the contributions from the “electrode effects” and/or the “porosity effects”. The “electrode effects” originated from the catalytic activity of Au electrodes. Conventionally the catalytic activity of Au electrodes is not considered to contribute to the resistance change of a chemiresistive sensor^32^. Even though there has been no unequivocal agreement so far about how the electrode materials affect the sensing behavior of a chemiresistive sensor^33^, there has been increasing evidence showing that different electrode metals react with analytes differently^18,34,35^. Some electrode metals, such as Au and palladium, can function as catalysts and contribute to the overall conductance and the sensitivity of the sensors^34,36–39^. “Porosity effects” originated from the creation of pores in the Au IDEs that led to increased Schottky contacts between the porous Au IDEs and the semiconducting SWCNTs. The Schottky contacts probably contributed more to the overall resistance change of a sensor than the bulk SWCNTs, which is supported by the increasing evidence showing that the interface between the semiconducting CNTs and the metal electrodes might play an important role in the performance of electronic devices such as gas sensors^20^ and transistors^40^. For our proof-of-concept porous Au-electroded sensor, the increased Schottky contacts between the porous Au IDEs and the semiconducting SWCNTs facilitated the around 5-fold increase in its sensitivity to the analyte DEEP.

There have been only a few reports in the literature describing the detection of DEEP vapour, most of which utilized mass spectrometry that featured high sensitivity and selectivity. For example, DEEP vapour has been detected with active capillary plasma ionization coupled to an ion trap mass spectrometer^41^ and with selected ion flow tube mass spectrometry (SIFT-MS)^42^ with a limit of detection of 0.15 ppb and 45 pptv, respectively. In addition to mass spectrometry, optical reflectivity spectroscopy has also been used to detect 135 ppm DEEP vapour^43^. To the best of our knowledge, the only chemiresistive DEEP sensor that has been reported so far is a proof-of-concept sensor previously developed by our group^4^. This sensor used the inherent properties of reduced graphene oxide to sense DEEP vapour with a relatively low sensitivity (a relative sensitivity of 5.2% was reached after exposure for 63 min to 2.0 ppm DEEP vapor, which is comparable to the sensing behavior of the dense Ag-electroded sensor reported in this work (Fig. 5)). Our proof-of-concept chemiresistive sensors are not as sensitive to DEEP vapour as mass spectrometry. However, mass spectrometry is usually associated with bulky equipment, lengthy sample preparation and solvent management. Our chemiresistive sensors, which are ultra-lightweight, flexible, miniature-sized and wearable, don’t require sample preparation or solvents and can be easily integrated into wireless sensing systems. Besides, our proof-of-concept sensors can be optimized and perform better. For example, for the sensing of G-type nerve agents or their simulants, our sensors, which used a low-cost, readily commercially available and structurally simple (with only one hexafluoroisopropanol group) selector (2-(2-hydroxy-1, 1, 1, 3, 3, 3-hexafluoropropyl)-1-naphthol), can perform better by using a polymeric selector (such as HC)^44^ containing multiple hexafluoroisopropanol groups. Such a polymeric selector is more efficient than a structurally simple selector in sensing a G-type nerve agent or a simulant, since each hexafluoroisopropanol group can absorb one target molecule by hydrogen bonding^44,45^.

The methods and techniques described in this work to increase the chemical stability of the electrodes and the Schottky contacts are facile and general, and can be applied to other semiconducting material-based sensors to enhance their sensitivity.

## Methods

### Functionalization of SWCNTs with a chemoselective compound

This process immobilized a hexafluoroisopropanol group-containing chemoselective compound (selector), which has been shown to bind the target analyte DEEP^4^, to semiconducting SWCNTs. A small sheet (15 mm × 15 mm) of semiconducting SWCNTs (IsoNanotubes-S. NanoIntegris Technologies, Inc., Boisbriand, Quebec, Canada) was immersed in dimethylformamide (DMF) and then sonicated slightly (10 seconds at power level 10) with a probe sonicator (Sonicator 3000, Misonix, Farmingdale, NY, USA). After a short centrifugation (4, 500 × g, 1 min) (5804R centrifuge, Eppendorf North America, Hauppauge, NY, USA), the resulting SWCNT particle suspension was incubated with a 5 mg/ml DMF-based solution of 2-(2-hydroxy-1, 1, 1, 3, 3, 3-hexafluoropropyl)-1-naphthol (Synquest Laboratories, Inc., Alachua, FL, USA) for 2 hours in an incubator shaker (25 °C, 90 rpm). After a centrifugation (4,500 × g, 5 min) to remove the unbound selector, the resulting SWCNT pellets were washed 3 times with DMF with a centrifugation (4,500 × g, 5 min) between each wash, and re-suspended in DMF. The suspension was further sonicated with the probe sonicator until a homogeneous DMF-based solution of SWCNT-selector complexes was obtained.

### Functionalization of Kapton^®^ substrate

**Figure 6:**
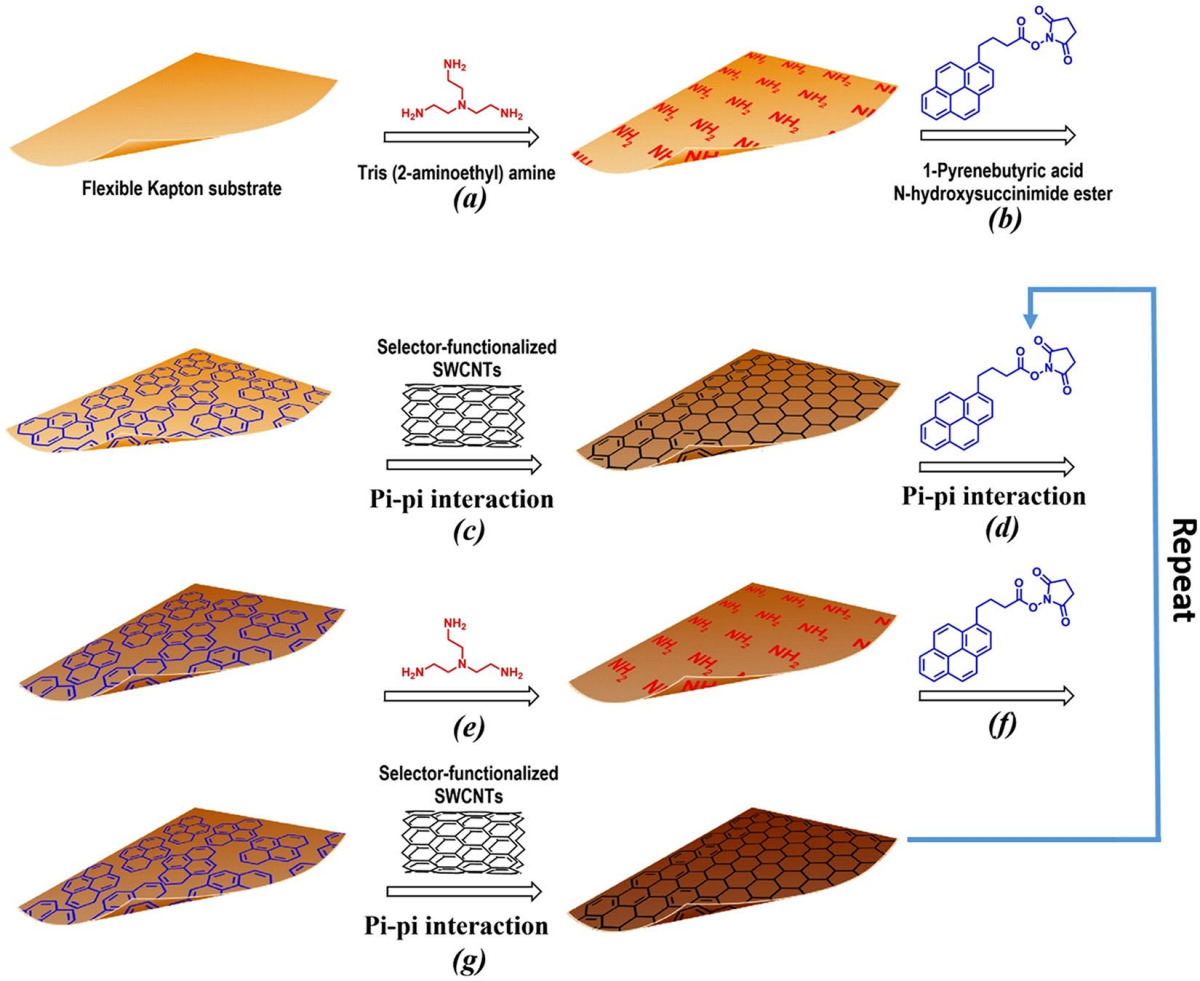
Layer-by-layer deposition of the SWCNTs and the chemoselective molecules on a flexible Kapton^®^ substrate.

**Figure 7:**
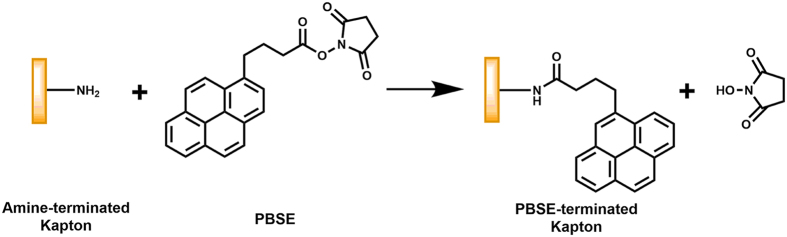
Reaction of the amine-terminated Kapton^®^ substrate with 1-pyrenebutyric acid N-hydroxysuccinimide ester (PBSE).

**Figure 8:**
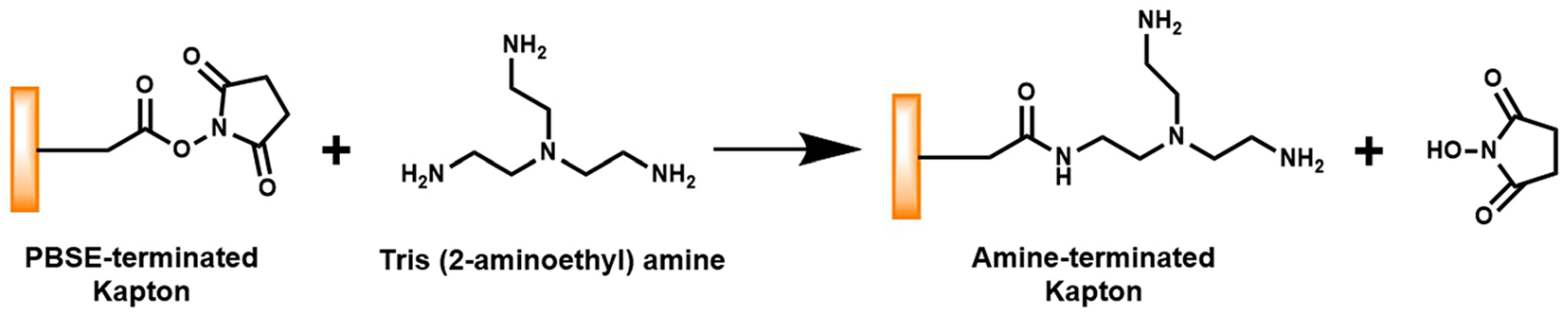
Reaction of the PBSE-terminated Kapton^®^ substrate with amine-containing tris (2-aminoethyl) amine.

A Kapton^®^ piece with dimensions of 95 mm × 95 mm was cut from a Kapton^®^ 500 HN sheet (Dupont, Wilmington, DE, USA). After sonicating first with a 10 g/L suspension of Powdered Precision Cleaner (Alconox, Inc., White Plains, NY, USA) in DI water for 10 min and then with acetone for 10 min in a 2510 Branson ultrasonic cleaner (Branson Ultrasonics, Danbury, CT, USA), followed by rinsing 3 times with DI water. The cleaned Kapton^®^ piece was then functionalized with the SWCNT-selector complexes in a layer-by-layer fashion. This wet chemical functionalization process, which uniformly deposited the SWCNT-selector complexes onto the surface-modified Kapton^®^ substrate, is depicted in Fig. 6. Briefly, the cleaned Kapton^®^ substrate was incubated with a DMF-based solution of 10 wt% tris (2-aminoethyl) amine for 1 hour to introduce surface amine groups to the substrate (Fig. 6 step (a)). After rinsing 3 times with DMF, the amine-terminated Kapton^®^ substrate was incubated with a DMF-based solution of 5 mg/ml 1-pyrenebutyric acid N-hydroxysuccinimide ester (PBSE) for 1 hour leading to the covalent bonding of PBSE to the substrate via Fig. 7 (Fig. 6 step (b)). After washing off the unbound species with DMF, the PBSE-functionalized Kapton^®^ substrate was incubated with the SWCNT-selector complex solution in DMF for 1 hour allowing for the binding of SWCNTs/selector to the Kapton^®^ substrate via π-π interaction (Fig. 6 step (c)). This completed the deposition of the first layer of selector-functionalized SWCNTs on the Kapton^®^ substrate. After washing 3 times with DMF, the resulting structure was incubated with the PBSE solution for 1 hour to deposit PBSE via π-π interaction (Fig. 6 step (d)). After washing 3 times with DMF, the PBSE-terminated structure was then incubated with a 10 wt% solution of tris (2-aminoethyl) amine in DMF for 1 hour to introduce surface amine groups via Fig. 8 (Fig. 6 step (e)). After washing 3 times with DMF, the resulting amine-terminated structure was then exposed to the PBSE solution for 1 hour to introduce surface PBSE via Fig. 7 (Fig. 6 step (f)). The resulting structure was then incubated with the SWCNT-selector solution to deposit the second layer of SWCNT-selector complexes on the Kapton^®^ substrate (Fig. 6 step (g)). Steps (d), (e), (f) and (g) were repeated 8 times (that is, a total of 10 layers of selector-functionalized semiconducting SWCNTs were deposited on the Kapton^®^ substrate).

### Inkjet-printing of Ag interdigitated electrodes

Ag IDEs, with both the width of every individual finger line and the spacing between two adjacent finger lines being 100 µm, were inkjet-printed with a drop-on-demand piezoelectric inkjet printer (DMP-2831, Fujifilm Dimatix, Inc., Santa Clara, CA, USA) for 5 passes on the functionalized Kapton^®^ HN substrate with a commercial Ag nanoparticle ink (Sun Chemical Corporation, Parsippany, NJ, USA), followed by drying in air at 120 °C for 3 h. After cooling down to the room temperature, some of the resulting sensor prototypes were immediately subjected to the Ag-to-Au conversion process described below.

### Conversion of inkjet-printed Ag IDEs into porous Au counterparts

A two-step process was performed to convert the inkjet-printed Ag IDEs into their highly porous Au counterparts under ambient conditions. The Ag IDE-Kapton^®^ structures (i.e., the sensors electroded with inkjet-printed Ag IDEs) were incubated with a 3 wt% solution of HAuCl_4_ (Sigma-Aldrich, St. Louis, MO, USA) in diluted HCl for 1 hour in an incubator shaker (25 °C, 90 rpm). After rinsing with DI water 3 times, the resulting structures were incubated with an aqueous solution of saturated NaCl^46,47^ (~360 g/L) in an incubator shaker (25 °C, 90 rpm) overnight to remove the side product AgCl. The resulting Au-Kapton^®^ structures (i.e., the sensors electroded with highly porous Au IDEs) were finally rinsed with DI water and dried with flowing air.

### Adhesion assessment

The adhesion between the Au or the Ag IDEs and the Kapton^®^ substrate was assessed by visual inspection and Scotch-tape peel tests. Visual inspection was performed from different angles while slowly bending, first in tension and then in compression, the Au- or Ag-electroded sensors to a radius of curvature of ~1 cm. Scotch-tape peel tests were performed by: (1) immobilizing (with the aid of double sided tape (3 M, St. Paul, MN, USA) and Quick Set^TM^ epoxy glue (Henkel Corporation, Rocky Hill, CT, USA)) the back side of the Ag- or Au-electroded sensors to a piece of plastic with a flat surface, (2) firmly sticking the adhesive side of a piece of Scotch^®^ magic tape (3 M) to the sensors, (3) peeling the tape off from the sensors, and (4) visually checking if the Ag or the Au IDEs on the sensors were still intact and if there were any Ag or Au residues left on the tape^48^.

### Gas sensing

A diethyl ethylphosphonate (DEEP) vapour (2.0 ppm) stream was generated from a DEEP permeation tube (KIN-TEK Laboratories, Inc., La Marque, TX, USA) in a FlexStream™ Gas Standards Generator (KIN-TEK Laboratories, Inc.) and carried by nitrogen (N_2_) gas with a flow rate of 500 sccm. Vapour sensing was performed with a homebuilt sensing system automated with NI LabVIEW software. In a typical DEEP sensing trial, two sensors, one electroded with the original inkjet-printed dense Ag IDEs and the other with the converted porous Au IDEs, were placed next to each other side by side in the sample chamber of the sensing system, and the resistance change with time of the sensors upon exposure to either carrier gas N_2_ or 2.0 ppm DEEP in N_2_ was automatically recorded by the system.

### Materials Characterization

Scanning electron microscopy (SEM) was conducted with a field emission scanning electron microscope (Leo 1530 FEG SEM, Carl Zeiss SMT Ltd., Cambridge, UK) equipped with an energy dispersive X-ray spectrometer (INCA EDS, Oxford Instruments, Bucks, UK). X-ray diffraction (XRD) analyses were performed with Cu Kα radiation using an X-Pert Pro Alpha-1 diffractometer equipped with an incident beam Johannsen monochromator and an Xcelerator linear detector (PANalytical, Almelo, The Netherlands). Scanned images were obtained from an Epson Perfection V370 Photo scanner.

## Electronic supplementary material

Supplementary Info
