# Author Correction: Sensitivity enhancement of flexible gas sensors via conversion of inkjet-printed silver electrodes into porous gold counterparts

**DOI:** 10.1038/s41598-018-24789-y

**Published:** 2018-04-16

**Authors:** Yunnan Fang, Mitra Akbari, Jimmy G. D. Hester, Lauri Sydänheimo, Leena Ukkonen, Manos M. Tentzeris

**Affiliations:** 1 School of Materials Science and Engineering, Georgia Institute of Technology, Atlanta, GA 30332-0245 USA; 2 Department of Electronics and Communications Engineering, Tampere University of Technology, 33101 Tampere, Finland; 3 School of Electrical and Computer Engineering, Georgia Institute of Technology, Atlanta, GA 30332-0250 USA

Correction to: *Scientific Reports* 10.1038/s41598-017-09174-5, published online 21 August 2017

Jimmy G. D. Hester was omitted from the author list in the original version of this Article. This has been corrected in the PDF and HTML versions of the Article, and in the accompanying supplementary material.

The Author Contributions section now reads:

Y.F., M.M.T. and J.G.D.H conceived the study. M.A. and Y.F. performed the inkjet-printing under the supervision of L.S. and L.U. Y.F. performed all other experiments and drafted the early version of the manuscript.

