# Supplementary material for: Sensitivity enhancement of flexible gas sensors via conversion of inkjet-printed silver electrodes into porous gold counterparts
Source: Sci Rep. 2017 Aug 21;7:8988. doi: 10.1038/s41598-017-09174-5 (PMC5566453; doi:10.1038/s41598-017-09174-5)
Supplement: Supplementary file 1 — Supplementary Info [file 41598_2017_9174_MOESM1_ESM.pdf]

Supplementary Information

Sensitivity enhancement of flexible gas sensors via conversion of inkjet-printed silver electrodes into porous gold counterparts

Yunnan Fang[1], Mitra Akbari[2], Jimmy G. D. Hester[3], Lauri Sydänheimo[2], Leena Ukkonen[2], Manos M. Tentzeris[3]

[1]School of Materials Science and Engineering, Georgia Institute of Technology, Atlanta, GA 30332-0245, USA

[2]Department of Electronics and Communications Engineering, Tampere University of Technology, 33101 Tampere, Finland

[3]School of Electrical and Computer Engineering, Georgia Institute of Technology, Atlanta, GA 30332-0250, USA

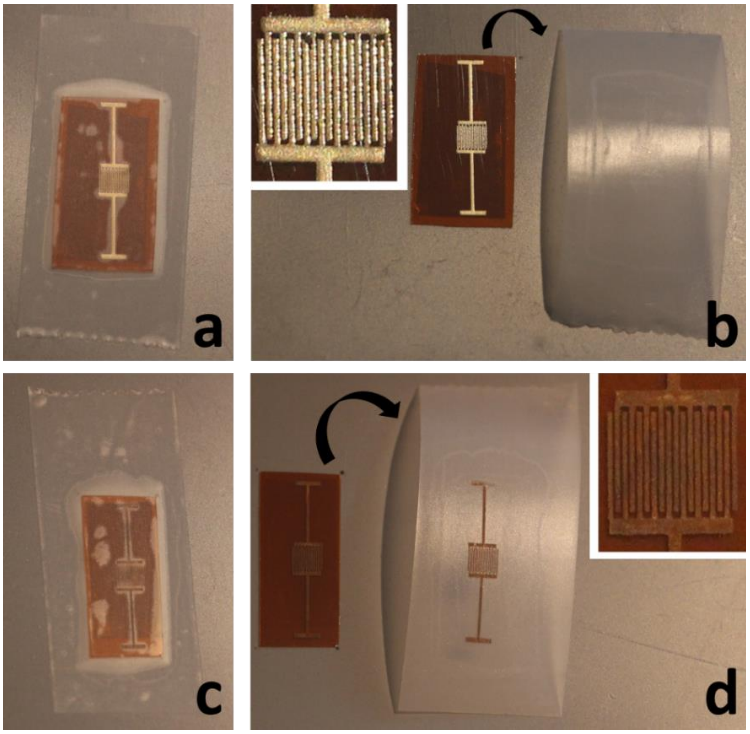

Supplementary Figure S1. Scotch-tape peel tests with the sensors electroded with the dense Ag IDEs and with the porous Au IDEs. a) and c) show optical images of the sensors electroded with the Ag IDEs and with the Au IDEs, respectively, that firmly stuck to the adhesive side of a piece of Scotch® magic tape. b) and d) show optical images of the sensors with the Ag IDEs and with the Au IDEs, respectively, after the tape has been peeled off. Insets in b) and c) show high magnification optical images of the IDE fingers on the Kapton® substrate shown in b) and c), respectively.
